# Relationships between pre-pandemic mental health, sociodemographic factors and health behaviours in older adults during the acute onset of COVID-19 in Australia: A descriptive analysis

**DOI:** 10.1371/journal.pone.0346787

**Published:** 2026-04-23

**Authors:** Nicole Lovato, Sarah L. Appleton, Amy C. Reynolds, Tiffany K. Gill, Sean Martin, Gary A. Wittert, Robert J. Adams

**Affiliations:** 1 Flinders Health and Medical Research Institute, Adelaide Institute for Sleep Health, Flinders University, Adelaide, South Australia, Australia; 2 Adelaide Institute for Sleep Health Clinic, Flinders University, Bedford Park, South Australia, Australia; 3 Adelaide Medical School, University of Adelaide, Adelaide, South Australia, Australia; 4 South Australian Health and Medical Research Institute, Adelaide, South Australia, Australia; 5 Australian Institute of Family Studies, Melbourne, Victoria, Australia; 6 Respiratory and Sleep Services, Southern Adelaide Local Health Network, Adelaide, South Australia, Australia; 7 Freemasons Centre for Male Health and Wellbeing, Adelaide Medical School, University of Adelaide, Adelaide, South Australia, Australia; Adelaide University, AUSTRALIA

## Abstract

**Objective:**

To gain a comprehensive understanding of associations between mental health symptoms and sociodemographic and health factors assessed during COVID-19 restrictions in existing, longitudinal community-based cohorts.

**Methods:**

Participants of The North West Adelaide Health Study (NWAHS, n = 982) and the Florey Adelaide Male Ageing Study (FAMAS, n = 338) in South Australia, undertook a COVID-19 impacts survey during October 2020-May 2021. The Centre for Epidemiologic Studies Depression Scale (score≥16;NWAHS) and the Beck Depression Inventory 1A (score≥13;FAMAS) were used to characterise mild-severe depressive symptoms. The Generalised Anxiety Disorder questionnaire was used to identify moderate-severe anxiety (score 10–21).

**Results:**

Of 1,320 participants (male n = 797), 62.4% (n = 824) were aged ≥65years (range 36−100 years), and 37.8% reported workforce participation at the time of the COVID-19 survey. Depressive and anxiety symptoms were observed for participants aged 35−54years (OR=1.92,95%CI = 1.01–3.67), financial stress (1.81,1.02–3.21), change in overall food intake (increase and decrease), social support none/sometimes(2.74,1.48–5.07), low control/mastery since COVID-19 (6.00,3.37–10.6) and poor sleep during restrictions (7.94,4.25–14.8), independent of previous depressive symptoms (8.30,1.9–13.2). Change in mental health status from pre-COVID to COVID-19 restriction was associated with sex (p = 0.013) and age (p < 0.001), such that females and younger participants (35−54yr) reported depressive symptoms at both times. Younger adults (35-54 yr) showed a higher prevalence of depressive symptoms only during COVID-19.

**Conclusions:**

Depressive and anxiety symptoms were consistent during COVID-19 relative to pre-COVID-19. Those with a history of depression, were more likely to report depressive and anxiety symptoms during COVID-19. Government-funded initiatives employed during future pandemics should consider tailored mental health and social support for vulnerable groups.

## Introduction

Economic and social impacts associated with COVID-19 led to substantial concerns about adverse effects on mental health, referred to in the media as a global mental health “pandemic” or “tsunami” [1,2]. However, a recent meta-analysis that included 137 studies from 134 cohorts around the world found no evidence of significant changes in the prevalence of mental health problems in the general population overall [1]. Small negative changes were seen in women or female participants for general mental health, anxiety and depression symptoms during the early part of the pandemic; and among older adults for depression symptoms. There was some evidence that mental health symptoms declined during the beginning of the pandemic but then stabilised to pre-pandemic levels in the second half of 2020 and into 2021 [3,4]. Among people with pre-existing mental health conditions there was negligible change in general mental health or depression symptoms.

Several studies have identified various health behaviours, and financial and occupational factors as risk factors for poor mental health during the pandemic [5]. A meta-analysis of 177 studies found a moderate correlation between sleep problems and depression and anxiety, particularly among those diagnosed with COVID-19 [6]. Some pre-pandemic predisposing factors include longitudinal associations of depressive and anxiety symptoms with social support, confidence in health care, and the stringency of the policy response during the pandemic (March 2020- April 2022) [7]. Lack of social support appears to act as a consistent predictor of depressive and anxiety symptoms, while the effect of the stringency of policy responses were dependent on the specific situations occurring within society.

Meta-analyses have identified that older adults experienced small increases in depressive symptoms during the pandemic [1], however significant gaps remain in the understanding of the specific factors associated with mental health outcomes in this population, particularly in the Australian context. Older adults represent a critical population due to their increased vulnerability to severe COVID-19 outcomes and the potential for social isolation due to social distancing recommendations. Further, most existing studies lack pre-pandemic baseline mental health data from the same individuals [3,4,7]. The current study leverages existing Australian cohorts with pre-pandemic baseline data [8–11], providing a unique opportunity to examine longitudinal mental health trajectories in older adults within the context of Australia’s distinct pandemic response.

The aim of this study was to use two existing, well characterised, community-based adult cohorts of largely older Australian adults to understand associations of mental health symptoms with sociodemographic and health factors assessed during COVID-19 restrictions in order to inform policy decisions and future interventions for mitigating the negative consequences of pandemics. This study addresses a critical gap in the literature by leveraging pre-pandemic baseline data [8–11] to examine longitudinal mental health trajectories in older Australians, a population that has been underrepresented in COVID-19 mental health research despite evidence of increased vulnerability to depression during the pandemic (1). Specifically, we aimed to:

1. Describe associations between mental health symptoms and perceived impact of COVID-19 on financial and occupational, health behaviour-related, and psychosocial factors in older adults in South Australia, and
2. Determine whether pre-pandemic mental health symptoms (2015−16) predicted mental health symptoms according to a survey conducted during the COVID-19 pandemic in this older adult population.

## Participants and methods

### Participants

The North West Adelaide Health Study (NWAHS) [8] and the Florey Adelaide Male Ageing Study (FAMAS) [9] are ongoing biomedical cohort studies of randomly selected adults from the north-west region of Adelaide, South Australia and have been described previously [10,11]. Both cohorts used the same sampling strategy for study recruitment. Households with a connected telephone and a telephone number listed in the Electronic White Pages (EWP) were randomly selected and the person aged at least 18 years who was last to have a birthday (last male person to have a birthday for FAMAS) was interviewed by telephone by trained health interviewers and asked to participate.

The baseline clinical assessments for the NWAHS (n = 4056, aged 18 years and over) and FAMAS (n = 1195 aged 35−80 years) occurred in 1999−2003 and 2002−2005 respectively. The NWAHS cohort underwent two further clinical follow-ups in 2004−2006 (n = 3206), and 2008−2010 (n = 2487) and the FAMAS cohort underwent a clinical follow-up in 2007−10 (n = 950). Postal follow-ups of the NWAHS (2015, n = 1560) and FAMAS (2015−16, n = 633) cohorts have also been conducted.

The COVID-19 impacts survey followed up participants of NWAHS (n = 982), and FAMAS (n = 338) using one of three methods: 1) paper-based questionnaires posted to participants, 2) using the secure, web-based software platform Research Electronic Data Capture (REDCap) [12,13], hosted at The University of Adelaide, and 3) over the phone with trained study staff. All measures were self-reported, and we used standardized tools and instruments used in previous waves of the cohorts. Data collection commenced October 6, 2020 (with the cumulative COVID-19 case numbers detected in South Australia since pandemic onset at 472). By December 23, 2020, 90% of follow-up surveys were completed, at which time cumulative COVID-19 case numbers were 566). Survey responses were accepted until May 30, 2021.

The Human Research Ethics Committees of the Central Adelaide Local Health Network (HREC/15/TQEH/127) and the University of Adelaide Human Research Ethics Committee (H-2020–109) approved the conduct of the surveys. The data were accessed for research purposes on 26 May 2023. The authors did not have access to information that could identify individual participants during or after data collection. All participants provided informed written consent.

### COVID context during the data collection period

On February 1^st^, 2020, the first case of COVID-19 was announced in South Australia. On March 11^th^, 2020, the South Australian Government announced stay-at-home recommendations and a $AUD350 million stimulus package to help support the South Australian economy and secure local jobs. Subsequent stimulus packages cumulatively totalled $AUD4bil (See Supplement 1 for further COVID-19 context). There was a total of 754 confirmed cases, from a total population of 1.77 million (42.6 cases per 100,000), from the start (October 2020) to the end (May 2021) of our data collection period.

### Mental health symptoms

Depressive symptoms were measured before the pandemic (2015–16), and during the pandemic (2020 COVID-19 survey). Symptoms of depression were determined using standard cut-offs according to the Centre for Epidemiologic Studies in Depression [14] (CES-D, ≥ 16) for NWAHS participants or the Beck Depression Inventory [15](BDI-1A, ≥ 13) for FAMAS participants. Depressive symptoms identified in the 2015–16 postal surveys were used to identify factors associated with participant reported depressive symptoms in 2020. Symptoms of anxiety were determined by the Generalised Anxiety Disorder questionnaire (GAD-7; [16]), which was dichotomised into no or mild anxiety (score ≤9) and moderate-severe anxiety (score of 10–21). GAD-7 data were not available in the 2020 COVID-19 survey, however of those reporting moderate to severe anxiety (n = 108) in 2015, 85% (n = 92) were captured in the at least mild depressive symptoms group in the 2020 COVID-19 survey. Collectively, this variable was captured as ‘mental health symptoms’.

### Demographic characteristics

Participants completed questions regarding standard demographic items, including age, marital status, education, their household’s level of financial stress, current income, and any workforce participation between January-March 2020 (just prior to the pandemic onset in Australia).

### Financial and occupational factors, health-related behaviour, and psychosocial factors

A range of questions were asked to determine any changes in work situation and financial position, nutrition, neighbourhood safety and social support, sleep quality, mastery, and behavioural risk factors for chronic disease, including alcohol consumption, physical activity and missed prescriptions. The items used to assess these factors and their response options are provided in Supplement 2.

### Statistical analysis

Analyses were conducted using IBM SPSS Statistics v28. Univariate differences in participant characteristics in relation to depression and anxiety symptoms (2020-21) were determined by Chi-square tests.

Binary logistic regression (BLR) analyses determined the best set of general and COVID-19 restriction related predictor variables (socio-demographic and health) for the presence of anxiety (at least moderate symptoms) and or depressive symptoms (mild-severe). The extent to which poor sleep troubled participants in general during COVID-19 restrictions was entered into the BLR model. Variables were entered into the model if univariate associations were present with a p value <0.25 [17].

In a final model, cross-sectional correlates of depressive and anxiety symptoms were additionally adjusted for previous depressive symptom levels determined in 2015−2016.

## Results

The 2020 COVID-19 survey was completed by 1,320 participants, of whom 60.4% were male, 62.4% were aged 65 years and older (range of 36–100 years), 62.2% were not in the workforce (mostly retired (55.5%)) and 69.2% were married or living with a partner (Table 1). Self-reported chronic disease risk factors were infrequent; 6% reporting current smoking, 76% consumed alcohol within the recommended guidelines and 63% of reported being physically active (150mins/week or more). Obesity (BMI > 30 kg/m^2^) was present in 32.5% of respondents.

**Table 1:** Participant characteristics during COVID follow-up.

Characteristic	n	%
**Sex**		
Male	797	60.4
Female	523	39.6
Total	1320	100.0
**Age**		
35-54y	158	12.0
55-64y	338	25.6
≥65y	824	62.4
Total	1320	100.0
**Marital status**		
Married/partner	896	69.2
Divorced, separated, widowed, never	398	30.8
Total	1294	100.0
**Highest education**		
High school or less	556	43.3
Trade, diploma, certificate	436	34.0
Bachelor degree or higher	291	22.7
Total	1283	100.0
**Household income ($AUD)**		
<20k	108	8.5
20-40k	268	21.1
40-80k	332	26.1
80-150k	239	18.8
≥150k	91	7.2
Refused/don’t know	232	18.3
Total	1270	100.0
Employment status (Jan-March 2020) **		
Working Full/Part-time	493	37.8
Retired/volunteer	723	55.5
Home duties/carer	44	3.4
Unemployed/unable to work	43	3.3
Total	1303	100.0
**Body mass index (Kg/m2)**		
<24.9	332	28.3
25.0-29.9	457	39.0
≥30	383	32.7
Total	1172	100.0
**Current smoking**		
Yes	66	5.0
No	1231	94.0
Occasionally	13	1.0
Total	1310	100.0
**Alcohol risk**		
10 drinks max/week and <5 on any day	990	75.7
>10/week OR <10/week but ≥5 drinks any day	317	24.3
Total	1307	100.0
**Physical activity**		
Sedentary	186	15.6
<150 min/week	258	21.6
≥150 min/week	750	62.8
Total	1194	100.0
Anxiety (GAD-7) or depressive symptoms (CESD/BDI) *		
No problem	1011	78.3
≥Moderate anxiety and or ≥mild depressive symptoms	281	21.7
Total	1292	100.0
**Previous depressive symptoms †**		
None	891	81.0
Mild-severe	209	19.0
Total	1100	100.0
**Mastery since Covid-19: “I have little control over things that happen to me”**		
Neutral	251	19.8
Agree	188	14.8
Disagree	828	65.4
Total	1267	100.0
**Can you get help from family, friends or neighbours when you need it?**		
None/some of time	173	13.3
Most of time	402	31.0
All of time	722	55.7
Total	1297	100.0
**During restrictions, to what extent has poor sleep troubled you in general?**		
Not at all	696	53.2
A little	443	33.9
Somewhat, much, very much	169	12.9
Total	1308	100.0
**Occupational/financial changes due to COVID-19 restrictions**		
**Change in financial position**		
A lot worse	53	4.1
Slightly worse	229	17.7
Slightly/a lot better	108	8.4
Stayed the same	869	67.3
Refused/don’t know	32	2.5
Total		
**Change in work status**		
Employed no change	262	53.5
Lost job/forced leave/lost volunteer role	42	8.6
Working usual hours (WFH**, different location, duties), more hours	113	23.1
Reduced hrs- WFH/usual place	66	13.5
Voluntarily left workforce	7	1.4
Total	490	100.0
**Disruption at work**		
Extreme/moderate	155	32.4
A little	142	29.7
Not at all/improved	181	37.9
Total	478	100.0

*Prescription medication was self-reported in n = 28 of those classified with no problem, and n = 32 in those with ≥moderate anxiety and or ≥mild depressive symptoms.

**WFH: work from home. † CES-D, ≥ 16 for NWAHS or BDI-1A, ≥ 13 for FAMAS participants in 2015−16.

**Fig 1:**
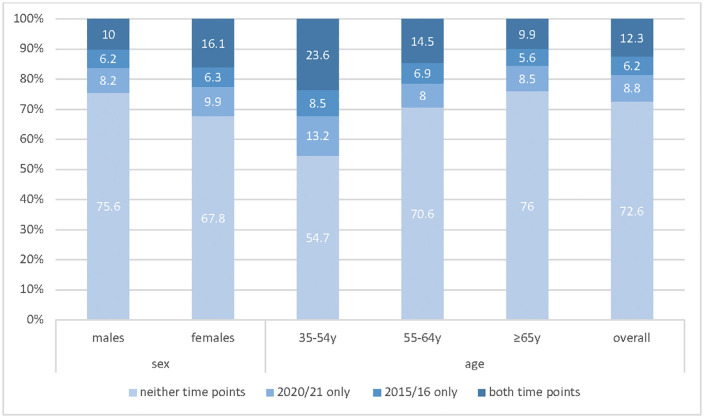
Prevalence of changing mental health status (depressive symptoms) between 2015/16 and the COVID-19 impacts survey in 2020−21 in South Australian adults.

Mental health symptoms were reported by 21.7% of the sample during the 2020 COVID-19 survey, with 19% of the sample reporting at least mild depressive symptoms in 2015−16 (Table 2 and Table 3). In 1,077 participants who had data in both 2015 and 2020, 72.6% (n = 782) were identified as never having depressive symptoms, 12.3% (n = 133) were symptomatic at both time points, 8.8% (n = 95) reported emergent depressive symptoms in the 2020 COVID-19 survey, and 6.2% (n = 67) reported symptoms only in 2015/16. A change in mental health symptoms was significantly associated with sex (p = 0.013) and age (p < 0.001), with females and younger participants (35−54yr olds) more frequently reporting depressive symptoms at both time points (Fig 1). Younger adults showed a higher prevalence of depressive symptoms only during the 2020 COVID-19 survey.

**Table 2:** Prevalence of anxiety and depressive symptoms (%, n) in relation to socio-demographic factors and COVID-19 restrictions related changes in occupation and financial position, overall and by sex.

	Males: no (%)	Males: no (n)	Males: yes (%)	Males: yes (n)	Males (p)	Females: no (%)	Females: no (n)	Females: yes (%)	Females: yes (n)	Females (p)	Overall: no (%)	Overall: no (n)	Overall: yes (%)	Overall: yes (n)	Overall (p)
**Sex**	–	–	–	–	–	–	–	–	–	–	–	–	–	–	0.002
males	–	–	–	–	–	–	–	–	–	–	81.2	634	18.8	147	–
females	–	–	–	–	–	–	–	–	–	–	73.8	377	26.2	134	–
**Age, years**	–	–	–	–	0.003	–	–	–	–	0.071	–	–	–	–	<0.001
35-54y	66.7	50	33.3	25	–	66.3	53	33.8	27	–	66.5	103	33.5	52	–
55-64y	81.5	159	18.5	36	–	70.3	97	29.7	41	–	76.9	256	23.1	77	–
65 + y	83.2	425	16.8	86	–	77.5	227	22.5	66	–	81.1	652	18.9	152	–
**Marital status**	–	–	–	–	<0.001	–	–	–	–	0.746	–	–	–	–	<0.001
married/partner	85.1	497	14.9	87	–	74.3	223	25.7	77	–	81.4	720	18.6	164	–
No partner	68.4	128	31.6	59	–	73.0	149	27.0	55	–	70.8	277	29.2	114	–
**Social support^†^**	–	–	–	–	<0.001	–	–	–	–	<0.001	–	–	–	–	<0.001
none/some of time	61.3	65	38.7	41	–	39.1	25	60.9	39	–	52.9	90	47.1	80	–
most of time	79.8	193	20.2	49	–	67.1	104	32.9	51	–	74.8	297	25.2	100	–
all of time	86.8	367	13.2	56	–	84.7	244	15.3	44	–	85.9	611	14.1	100	–
**Highest education**	–	–	–	–	0.516	–	–	–	–	0.004	–	–	–	–	0.681
High school or less	82.1	239	17.9	52	–	74.4	189	25.6	65	–	78.5	428	21.5	117	–
Trade, diploma, certificate	82.1	261	17.9	57	–	63.2	72	36.8	42	–	77.1	333	22.9	99	–
Bachelor degree or higher	78.1	121	21.9	34	–	81.8	108	18.2	24	–	79.8	229	20.2	58	–
**Financial stress**	–	–	–	–	<0.001	–	–	–	–	<0.001	–	–	–	–	<0.001
spend≥ earnings	63.3	69	36.7	40	–	54.9	56	45.1	46	–	59.2	125	40.8	86	–
saves some or a lot	83.2	476	16.8	96	–	77.4	254	22.6	74	–	81.1	730	18.9	170	–
refused/don’t know	89.0	89	11.0	11	–	82.7	67	17.3	14	–	86.2	156	13.8	25	–
**Household income, ($AUD)**	–	–	–	–	0.679	–	–	–	–	0.396	–	–	–	–	0.146
<$20k	77.6	45	22.4	13	–	62.0	31	38.0	19	–	70.4	76	29.6	32	–
$20-40k	79.0	124	21.0	33	–	72.0	77	28.0	30	–	76.1	201	23.9	63	–
$40-80k	82.6	181	17.4	38	–	75.9	85	24.1	27	–	80.4	266	19.6	65	–
$80-150k	85.1	137	14.9	24	–	76.3	58	23.7	18	–	82.3	195	17.7	42	–
>$150k	80.0	44	20.0	11	–	80.6	29	19.4	7	–	80.2	73	19.8	18	–
Refused/don’t know	79.6	86	20.4	22	–	74.6	85	25.4	29	–	77.0	171	23.0	51	–
**Employment status (Jan-March 2020)**	–	–	–	–	<0.001	–	–	–	–	0.088	–	–	–	–	<0.001
Working Full/Part-time	80.1	234	19.9	58	–	75.3	149	24.7	49	–	78.2	383	21.8	107	–
Retired/volunteer	84.2	385	15.8	72	–	75.9	192	24.1	61	–	81.3	577	18.7	133	–
Home duties/carer	50.0	3	50.0	3	–	64.9	24	35.1	13	–	62.8	27	37.2	16	–
Unemployed/unable to work	31.6	6	68.4	13	–	54.5	12	45.5	10	–	43.9	18	56.1	23	–
**COVID restriction related change in work status if workforce participants**	–	–	–	–	0.193	–	–	–	–	0.178	–	–	–	–	0.173
No change	84.0	131	16.0	25	–	76.9	80	23.1	24	–	81.2	211	18.8	49	–
Lost job/forced leave/lost volunteer role	73.7	14	26.3	5	–	72.7	16	27.3	6	–	73.2	30	26.8	11	–
Working usual hours (WFH*, different location, duties), more hours	70.8	46	29.2	19	–	75.0	36	25.0	12	–	72.6	82	27.4	31	–
Reduced hrs- WFH/usual place	84.4	38	15.6	7	–	76.2	16	23.8	5	–	81.8	54	18.2	12	–
Voluntarily left workforce	80.0	4	20.0	1	–	0.0	0	100.0	2	–	57.1	4	42.9	3	–
**Change in financial position due to COVID-19**	–	–	–	–	0.042	–	–	–	–	0.223	–	–	–	–	0.034
slightly or a lot worse	76.8	142	23.2	43	–	68.8	64	31.2	29	–	74.1	206	25.9	72	–
no change, slightly, a lot better	83.4	482	16.6	96	–	75.0	291	25.0	97	–	80.0	773	20.0	193	–
**Disruption at work**	–	–	–	–	0.095	–	–	–	–	0.035	–	–	–	–	0.004
extreme/mod disruption	72.9	78	27.1	29	–	64.8	46	35.2	25	–	69.7	124	30.3	54	–
a little	79.8	79	20.2	20	–	68.6	48	31.4	22	–	75.1	127	24.9	42	–
not at all/improved	83.4	161	16.6	32	–	81.4	83	18.6	19	–	82.7	244	17.3	51	–

WFH: work from home.

^†^ Can you get help from family, friends or neighbours when you need it?

CES-D, ≥ 16 for NWAHS or BDI-1A, ≥ 13 for FAMAS participants in 2015−16.

**Table 3:** Prevalence of anxiety and depressive symptoms (%, n) in relation to health behaviours and psychosocial factors overall and by sex.

	Males					Females					Overall				
	no		yes		p-value	no		yes		p-value	no		yes		p-value
	%	n	%	n		%	n	%	n		%	n	%	n	
**Previous depressive symptoms†**					<0.001					<0.001					<0.001
no symptoms	90.3	500	9.7	54		87.3	282	12.7	41		89.2	782	10.8	95	
mild, moderate, severe	38.3	41	61.7	66		28.0	26	72.0	67		33.5	67	66.5	133	
**Body mass Index, Kg/m2**					0.002					0.022					<0.001
<25	89.3	150	10.7	18		82.1	128	17.9	28		85.8	278	14.2	46	
25-29	83.3	265	16.7	53		72.2	96	27.8	37		80.0	361	20.0	90	
≥ 30	75.8	169	24.2	54		68.8	106	31.2	48		72.9	275	27.1	102	
**Current smoker**					0.003					0.025					<0.001
Yes	68.4	26	31.6	12		51.9	14	48.1	13		61.5	40	38.5	25	
No	82.6	602	17.4	127		75.2	358	24.8	118		79.7	960	20.3	245	
Occasionally	42.9	3	57.1	4		66.7	4	33.3	2		53.8	7	46.2	6	
**Alcohol use**					0.474					0.43					0.665
10 drinks max/week and <5 on any day	81.8	444	18.2	99		74.2	317	25.8	110		78.5	761	21.5	209	
>10/week OR <10/week but ≥ 5 drinks any day	79.6	183	20.4	47		70.0	56	30.0	24		77.1	239	22.9	71	
**Physical activity**					0.001					0.006					<0.001
sedentary	71.2	79	28.8	32		62.9	44	37.1	26		68.0	123	32.0	58	
<150 min/week	78.9	105	21.1	28		71.7	86	28.3	34		75.5	191	24.5	62	
≥ 150min/week	85.5	408	14.5	69		80.3	212	19.7	52		83.7	620	16.3	121	
**Missed or delayed getting regular prescription medication as a result of COVID19 restrictions**					0.009					<0.001					<0.001
Yes	67.9	36	32.1	17		48.8	21	51.2	22		59.4	57	40.6	39	
No	82.3	569	17.7	122		76.6	340	23.4	104		80.1	909	19.9	226	
**Diet change since COVID19 restrictions began (March 2020)**					<0.001					<0.001					<0.001
< before	69.4	43	30.6	19		54.1	20	45.9	17		63.6	63	36.4	36	
same	84.2	542	15.8	102		78.7	303	21.3	82		82.1	845	17.9	184	
> before	65.7	46	34.3	24		60.9	53	39.1	34		63.1	99	36.9	58	
**Mastery: Since Covid-19 “I have little control over the things that happen to me”**					<0.001					<0.001					<0.001
neutral	68.0	104	32.0	49		65.2	60	34.8	32		66.9	164	33.1	81	
agree	52.6	51	47.4	46		37.1	33	62.9	56		45.2	84	54.8	102	
disagree	90.5	455	9.5	48		85.8	273	14.2	45		88.7	728	11.3	93	
**Confidence filling medical forms by yourself**					0.008					0.046					0.002
not at all/little/moderate	73.9	113	26.1	40		63.9	46	36.1	26		70.7	159	29.3	66	
very/extremely	83.2	509	16.8	103		75.1	323	24.9	107		79.8	832	20.2	210	
**Poor sleep: “Troubled you in general?” Since Covid-19**					<0.001					<0.001					<0.001
not at all	93.1	417	6.9	31		89.7	217	10.3	25		91.9	634	8.1	56	
a little	73.2	180	26.8	66		69.8	132	30.2	57		71.7	312	28.3	123	
somewhat, much, very much	41.5	34	58.5	48		33.3	26	66.7	52		37.5	60	62.5	100	

^†^ CES-D, ≥ 16 for NWAHS or BDI-1A, ≥ 13 for FAMAS participants in 2015−16.

Over half of the participants in the 2020 COVID-19 survey reported being able to get help from family, friends or neighbours if needed all of the time (56%), and 65% of participants disagreed with the statement that since COVID-19 “I have little control over things that happen to me”. During restrictions, just over half (53.2%) reported that they were not troubled by poor sleep. Most respondents (75.7%) reported their financial position improved or remained the same, but 4.1% reported it had worsened a lot with COVID-19. Of those working, just over half (53.5%) indicated that there were no changes to work status for with COVID-19.

Previously reported depressive symptoms were a strong predictor of mental health symptoms during COVID-19. In men, financial stress (regardless of employment status) was also strongly associated with mental health symptoms (see Supplementary Table 3). More broadly, mental health symptoms during COVID-19 were significantly associated with age (35−54 years), current smoking, reporting low levels of social support, change (both increases and decreases) in food intake, subjective poor sleep, missing medication prescriptions which needed to be filled, reporting a sense of little control over things that happen to oneself (Table 4). These associations persisted after adjustment for previous depressive symptoms in 2015−16, which were also strongly associated with mental health symptoms during the 2020 COVID-19 survey.

**Table 4:** Logistic regression analysis of factors associated with depression and anxiety symptoms at follow up.

	Model 1		Model 2	
	OR (95% CI)		OR (95% CI)	p-value
**Age, yr**				
35-54y	2.31 (1.27-4.21)	0.006	1.92 (1.01-3.67)	0.048
55-64	1.04 (0.64-1.69)	0.86	0.93 (0.55-1.57)	0.78
≥65	1.00		1.00	
**Sex**				
female	1.23 (0.82-1.83)	0.32	1.27 (0.82-1.97)	0.28
male	1.00		1.00	
**Overall food intake/day since restrictions began**				
<before	2.22 (1.11-4.44)	0.025	2.55 (1.24-5.23)	0.005
>before	2.00 (1.16-3.39)	0.012	2.01 (1.13-3.58)	0.018
same	1.00		1.00	
**Smoking**				
yes/some days	2.86 (1.27-6.44)	0.011	2.05 (0.83-5.08)	0.12
non-smoker	1.00		1.00	
**Missed/delayed getting regular prescription medication?**				
yes	2.20 (1.16-4.17)	0.015	1.55 (0.76-3.16)	0.23
no	1.00		1.00	
**Financial stress**				
Spending equivalent to/exceeds earnings	1.63 (0.97-2.74)	0.063	1.81 (1.02-3.21)	0.044
don’t know/refused	0.68 (0.34-1.36)	0.28	0.88 (0.43-1.79)	0.73
saves a lot/ bit left over saved or spent	1.00		1.00	
**Confident filling medical forms alone**				
not at all/a little/moderate	1.22 (0.74-2.03)	0.44	1.19 (0.68-2.09)	0.44
extremely/very	1.00		1.00	
**Social support (Can get help from family, friends or neighbours when needed)**				
none/some of time	3.06 (1.74-5.38)	<0.001	2.74 (1.48-5.07)	0.001
most of time	1.89 (1.22-2.92)	0.004	1.79 (1.11-2.86)	0.016
all of the time	1.00		1.00	
**Mastery (since covid, have little control over things that happen to me)**				
neutral	3.90 (2.44-6.23)	<0.001	3.44 (2.07-5.70)	<0.001
agree	8.12 (4.81-13.7)	<0.001	6.00 (3.37-10.6)	<0.001
disagree	1.00		1.00	
**Sleep-During the restrictions, to what extent has poor sleep, troubled you in general?**				
somewhat/much/very much	14.0 (7.86-24.8)	<0.001	3.44 (2.11-5.61)	<0.001
a little	4.67 (2.97-7.34)	<0.001	7.94 (4.25-14.8)	<0.001
not at all	1.00		1.00	
**Previous depressive symptoms (2015−16)†**				
At least mild			8.29 (5.19-13.2)	<0.00
no symptoms			1.00	

^†^ CES-D ≥ 16 for NWAHS or BDI-1A, ≥ 13 for FAMAS participants.

Model 2: model 1 additionally adjusted for previous depressive symptoms.

## Conclusions

In middle-aged and older adults, pre-existing depressive symptoms are predictive of mental health symptoms during the COVID-19 pandemic. This was observed in the context of a tightly controlled pandemic environment, with modest active cases at the time of survey. Females and early-middle aged adults more frequently reported depressive symptoms both before and during the COVID-19 pandemic. Other predictors also included lower levels of self-reported social support and feeling a sense of having little control over things that happen in an individual’s life. Together, these findings align with previous findings [5–7] and highlight the importance of mental health support for adults with pre-existing mental health conditions in pandemic or major event contexts, and the importance of considering intervention for those with lower levels of social support.

The generalisability of our findings needs to be considered in the context of the relatively low COVID-19 case numbers in Australia, the brief lockdown periods during the period of data collection, and the substantial financial support provided to workers in Australia. Together these may have reduced the impact of the pandemic on mental health. Our study sample is not representative of the Australian population where (based upon 2021 Census data [18]), adults aged 55 years or higher comprise 29% of the population and females comprise 50.7%. This is to be expected however, as the FAMAS cohort recruited only males aged at least 35 years. Both cohorts were established in the early 2000s and there has been loss to follow-up and mortality without sample replacement. Despite this, it is a strength of our study that our described prevalence of anxiety and/or depressive symptoms is similar to that from the 2020–2022 National Study of Mental Health and Wellbeing [19] in both the South Australian population aged 16–85 (21.6% had a 12-month mental disorder), and the Australian population in males (18%) and females (25%). Our findings are also consistent with the well known phenomenon that females report higher rates of mental health symptoms than males. The cross-sectional nature of the pre-pandemic data does not allow for causal inferences to be drawn. The reliance on self-report measures introduces the potential for recall bias, which may affect the accuracy of reported symptoms and experiences. The identification of specific risk factors and population groups at risk of poor mental health in a pandemic enables targeted interventions using existing systems (e.g., Telecross REDi) and informs policy development. Initiatives aimed at promoting good quality sleep and improved accessibility to sleep disorder treatment, particularly for those at working age, may also be warranted.

## Supporting information

S1 File Announcements made by the South Australian Government in relation to health and financial well-being. (DOCX)

S2 File Complete questionnaire list. (DOCX)

S3 File Sex-specific logistic regression analysis of factors associated with depression and anxiety symptoms at follow up. (DOCX)
