# Supplementary material for: Relationships between pre-pandemic mental health, sociodemographic factors and health behaviours in older adults during the acute onset of COVID-19 in Australia: A descriptive analysis
Source: PLoS One. 2026 Apr 23;21(4):e0346787. doi: 10.1371/journal.pone.0346787 (PMC13105359; doi:10.1371/journal.pone.0346787)
Supplement: S1 File — (DOCX) [file pone.0346787.s001.docx]

#### **S1. Announcements made by the South Australian Government in relation to health and financial well-being** (https://www.aph.gov.au/About_Parliament/Parliamentary_departments/Parliamentary_Library/pubs/rp/rp2021/Chronologies/COVID-19StateTerritoryGovernmentAnnouncements#_Toc52275798)

| **Milestones** | **Details** | **Source documents** |
| --- | --- | --- |
| 1 February 2020 | The SA Government announced the first two confirmed cases of coronavirus in SA and advised anyone who had visited mainland China or had had contact with a confirmed case to isolate for 14 days. | S Marshall (SA Premier), Premier’s statement on coronavirus, media release, 1 February 2020. |
| 10 March 2020 | The Premier declared that the nation’s first drive-through COVID-19 testing facility would open in SA the next day. | S Marshall (SA Premier), [Australia’s first drive-through COVID-19 testing clinic,](https://www.premier.sa.gov.au/news/media-releases/news/australias-first-drive-through-covid-19-testing-clinic) media release, 10 March 2020. |
| 11 March 2020 | The SA Government announced a $350 million stimulus package to help ‘drive the South Australian economy and secure local jobs’ in response to the coronavirus crisis. | S Marshall (SA Premier), [Unprecedented response and economic stimulus to drive SA jobs, economy in wake of bushfires, coronavirus,](https://www.premier.sa.gov.au/news/media-releases/news/unprecedented-response-and-economic-stimulus-to-drive-sa-jobs,-economy-in-wake-of-bushfires,-coronavirus2) media release, 11 March 2020. |
| 17 March 2020 | The SA Government announced a series of COVID-19 regional clinics to ensure widespread access to testing in metropolitan and regional areas. | S Marshall (SA Premier) and S Wade (SA Minister for Health and Wellbeing), [South Australia’s first regional COVID-19 clinic opens,](https://www.premier.sa.gov.au/news/media-releases/news/south-australias-first-regional-covid-19-clinic-opens2) media release, 17 March 2020. |
| 18 March 2020 | The SA Government and South Australia Police (SAPOL) announced that anyone arriving at Adelaide Airport on an international flight would be given a direction by Australian Border Force outlining their obligation to self-isolate at home for 14 days, with SAPOL conducting spot-checks to ensure they were abiding by the Direction. | S Marshall (SA Premier), [Stronger powers to enforce COVID-19 self-isolation,](https://www.premier.sa.gov.au/news/media-releases/news/stronger-powers-to-enforce-covid-19-self-isolation) media release, 18 March 2020. |
| 19 March 2020 | The SA Government granted an exemption to supermarkets under the Shop Trading Hours Act 1977 to allow 24-hour weekday trading to assist in social distancing measures. | R Lucas (SA Treasurer), [Supermarkets granted 24-hour weekday trade option and extended weekends in response to coronavirus,](https://www.premier.sa.gov.au/news/media-releases/news/supermarkets-granted-24-hour-weekday-trade-option-and-extended-weekends-in-response-to-coronavirus) media release, 19 March 2020. |
| 22 March 2020 | The Premier announced the closure of SA borders from 24 March, with anyone entering from overseas or other states or territories required to self-isolate for 14 days.  South Australia’s borders closed to international and interstate travel. The first COVID-related death was recorded on April 7th, 2020. Despite “stay at home” recommendations from early March 2020, there was no official lockdown in South Australia until November of 2020, which lasted for 6 days. During 2020, there were reductions in travel, workplace activity, retail, and recreation, including parks. The perceptions of residents meant they stayed at home, but much less so than people residing interstate or overseas. | S Marshall (SA Premier),[South Australia’s borders to close,](https://www.premier.sa.gov.au/news/media-releases/news/south-australias-borders-to-close) media release, 22 March 2020. |
| 24 March 2020 | The SA Government announced the opening of a second metropolitan COVID-19 drive-through testing facility to increase testing options for South Australians. | S Marshall (SA Premier), [Second metropolitan COVID-19 testing drive through opens,](https://www.premier.sa.gov.au/news/media-releases/news/second-metropolitan-covid-19-testing-drive-through-opens) media release, 24 March 2020. |
| 26 March 2020 | The SA Government announced a $650 million Jobs Rescue Package with two parts:   - the $300 million Business and Jobs Support Fund to support individual businesses and industry sectors directly affected by coronavirus and   the $250 million Community and Jobs Support Fund to support community organisations, sporting, arts and recreational bodies and non-profit organisations, and to help with training of South Australians seeking new skills and employment. | S Marshall (SA Premier), [$1 billion stimulus package to save SA jobs, businesses,](https://www.premier.sa.gov.au/news/media-releases/news/$1-billion-stimulus-package-to-save-sa-jobs,-businesses) media release, 26 March 2020. |
| 30 March 2020 | The SA Government encouraged vulnerable South Australians to register for the Red Cross Telecross REDi service. The service offers free daily calls to check on the welfare of vulnerable South Australians. | S Marshall (SA Premier), [New COVID-19 welfare calls activated for our most vulnerable,](https://www.premier.sa.gov.au/news/media-releases/news/new-covid-19-welfare-calls-activated-for-our-most-vulnerable) media release, 30 March 2020. |
| 31 March 2020 | The SA Government rolled out the South Australian Virtual Support Network to provide a localised service for South Australians who need additional mental health support during COVID-19. The Virtual Support Network included a mental health support line staffed by Lifeline counsellors to assist people with the increased anxiety, fear and distress the pandemic created. | S Marshall (SA Premier) and S Wade (SA Minister for Health and Wellbeing), [SA COVID-19 Mental Health Support Line,](https://www.premier.sa.gov.au/news/media-releases/news/sa-covid-19-mental-health-support-line) media release, 31 March 2020. |
| 1 April 2020 | The Minister for Health and Wellbeing announced changes under an emergency measure that would enable South Australians in need of an emergency supply of medicines during the coronavirus pandemic to temporarily seek their medication without a prescription, to minimise the burden of the pandemic on vulnerable South Australians.  Changes under an emergency measure enabled South Australians in need of an emergency supply of medicines during the coronavirus pandemic to temporarily seek their medication without a prescription, to minimise the burden of the pandemic on vulnerable South Australians. | S Marshall (SA Premier) and S Wade (SA Minister for Health and Wellbeing), [Providing essential medicines to South Australians,](https://www.premier.sa.gov.au/news/media-releases/news/providing-essential-medicines-to-south-australians) media release, 1 April 2020. |
| 3 April 2020 | The SA Government launched a flexible learning platform which complemented the Our Learning SA website and enabled teachers to create their own individual online learning spaces to deliver lessons and content to students in the classroom and at home. | J Gardner (SA Minister for Education), [Virtual classrooms to assist student learning,](https://www.premier.sa.gov.au/news/media-releases/news/virtual-classrooms-to-assist-student-learning) media release, 3 April 2020. |
| 4 April 2020 | The SA Premier urged South Australians to stay home during Easter and to follow the advice of health experts to limit the spread of COVID-19. | S Marshall (SA Premier), [For the health and wellbeing of all South Australians—please stay home this Easter,](https://www.premier.sa.gov.au/news/media-releases/news/for-the-health-and-wellbeing-of-all-south-australians-please-stay-home-this-easter) media release, 4 April 2020. |
|  | The SA Government announced a $1.6 million funding boost to help vulnerable South Australians, including:   - an additional $500,000 for the food relief sector - $800,000 for charities so they can give emergency relief to vulnerable South Australians, including supermarket and pharmacy vouchers and help with cost of living pressures - $185,000 to the Salvation Army, which provides the Affordable SA Helpline and National Debt Helpline SA branch and   $144,000 to existing providers within the Statewide Financial Counselling Program. | M Lensink (SA Minister for Human Services), [$1.6 million funding boost to help vulnerable,](https://www.premier.sa.gov.au/news/media-releases/news/$1.6-million-funding-boost-to-help-vulnerable) media release, 5 April 2020. |
| 7 April 2020 | - SA Health announced the first COVID-19 related death in SA. | SA Health, [COVID-19 update,](https://www.sahealth.sa.gov.au/wps/wcm/connect/public+content/sa+health+internet/about+us/news+and+media/all+media+releases/covid-19++update+7+april+2020) media release, 7 April 2020. |
| 7 April 2020 | The new COVID-19 Relief Call Centre was activated by the SA Government to support people requiring personal hardship assistance, food relief, emotional and wellbeing support, and short-term COVID-19 accommodation advice (for those unable to quarantine at home). | M Lensink (SA Minister for Human Services), [New, dedicated relief hotline activated to support South Australians in need,](https://www.premier.sa.gov.au/news/media-releases/news/new,-dedicated-relief-hotline-activated-to-support-south-australians-in-need) media release, 9 April 2020. |
|  | Tens of thousands of South Australian small businesses and NGOs were offered a one-off payment of $10,000 emergency cash in response to the coronavirus crisis. The grants were funded by the SA Government’s $650 million Jobs Rescue Package. | S Marshall (SA Premier), [$10,000 emergency cash grants for small businesses impacted by COVID-19,](https://www.premier.sa.gov.au/news/media-releases/news/$10,000-emergency-cash-grants-for-small-businesses-impacted-by-covid-19) media release, 9 April 2020. |
| 22 April 2020 | The SA Government announced a total of $7.8 million to be paid to eligible South Australians receiving the Centrelink Jobseeker Payment in the form of a one-off payment of $500 to help with everyday expenses. | M Lensink (SA Minister for Human Services), [$7.8 million cash boost to help with cost of living,](https://www.premier.sa.gov.au/news/media-releases/news/$7.8-million-cash-boost-to-help-with-cost-of-living) media release, 22 April 2020. |
| 23 April 2020 | The SA Minister for Health and Wellbeing praised the chemo@home program which allows eligible patients undergoing cancer treatment at the Royal Adelaide Hospital to undergo treatment at home, minimising the need for them to attend the hospital during the pandemic. | S Wade (SA Minister for Health and Wellbeing), [Protecting vulnerable South Australians from COVID-19](https://www.premier.sa.gov.au/news/media-releases/news/protecting-vulnerable-south-australians-from-covid-19), media release, 23 April 2020. |
| 26 April 2020 | The SA Government urged South Australians with medical issues not to delay seeking medical advice for fear of contracting COVID-19 after a concerning reduction in presentations to emergency, ambulance call-outs and GP visits. | S Wade (SA Minister for Health and Wellbeing), [Our health system is for all South Australians,](https://www.premier.sa.gov.au/news/media-releases/news/our-health-system-is-for-all-south-australians) media release, 26 April 2020. |
| 29 April 2020 | The SA Government committed $3.35 million towards COVID-19 Support Grants to support organisations that provide assistance to vulnerable and disadvantaged South Australians. Each organisation could apply for up to $10,000. | M Lensink (SA Minister for Human Services), [New, COVID-19 Support Grants of up to $10,000 available,](https://www.premier.sa.gov.au/news/media-releases/news/new,-covid-19-support-grants-of-up-to-$10,000-available) media release, 29 April 2020. |
| 30 April 2020 | The SA Government announced the COVID-19 GP Assessment Team and nurse-led SA Health Remote Monitoring Service to assist patients with COVID-19 who are isolating at home. | S Wade (SA Minister for Health and Wellbeing), [GP and nurse teams support COVID-19 patients at home,](https://www.premier.sa.gov.au/news/media-releases/news/gp-and-nurse-teams-support-covid-19-patients-at-home) media release, 30 April 2020. |
| 2 May 2020 | The Minister for Health and Wellbeing noted a significant increase in telehealth services during COVID-19, with an increase of nearly 150 per cent in the period from February to April this year compared to last year. | S Wade (SA Minister for Health and Wellbeing), [Embracing technology to provide better health services during COVID,](https://www.premier.sa.gov.au/news/media-releases/news/embracing-technology-to-provide-better-health-services-during-covid) media release, 2 May 2020. |
| 9 May 2020 | SA Health announced the release of the SA roadmap for easing COVID-19 restrictions, with Step 1 coming into effect on 11 May 2020. | SA Health[, COVID-19 update 9 May 2020,](https://www.sahealth.sa.gov.au/wps/wcm/connect/public+content/sa+health+internet/about+us/news+and+media/all+media+releases/covid-19+update+9+may++2020) media release, 9 May 2020. |
| 14 May 2020 | The Minister for Innovation and Skills reported results from the Australian Bureau of Statistics Labour Force Survey which showed unemployment in SA increased from 6.3 per cent in March to 7.2 per cent in April 2020. This increase was thought to be due to, but not fully reflective of, the effects of COVID-19 on the labour market. | D Pisoni (SA Minister for Innovation and Skills), [SA unemployment rate increase in line with increase nationally,](https://www.premier.sa.gov.au/news/media-releases/news/sa-unemployment-rate-increase-in-line-with-increase-nationally) media release, 14 May 2020. |
| 25 May 2020 | The Premier announced accelerated changes to COVID-19 restrictions, with pubs, gyms, cinemas, places of worship, beauty salons and other sites to be allowed up to 80 people from 1 June as long as they comply with social distancing safeguards. | S Marshall (SA Premier), [Fast-tracking economic recovery,](https://www.premier.sa.gov.au/news/media-releases/news/fast-tracking-economic-recovery) media release, 25 May 2020. |
| 1 June 2020 | SA Health announced the commencement of Step 2 of the SA [Roadmap for Easing COVID-19 Restrictions](http://www.covid-19.sa.gov.au/recovery). | SA Health, [COVID-19 update 1 June,](https://www.sahealth.sa.gov.au/wps/wcm/connect/public+content/sa+health+internet/about+us/news+and+media/all+media+releases/covid-19+update+1+june) media release, 1 June 2020. |
| 5 June 2020 | A new scheme worth approximately $10 million was announced by the SA Government to provide rent relief to eligible South Australians through a $1,000 grant to their landlords. The scheme was part of the Government’s $1 billion economic stimulus package to support local jobs, businesses and livelihoods during COVID-19. | R Lucas (SA Treasurer), [$1000 rent relief grants to further support residential tenants,](https://www.premier.sa.gov.au/news/media-releases/news/$1000-rent-relief-grants-to-further-support-residential-tenants) media release, 5 June 2020. |
| 29 June 2020 | SA Health announced the commencement of Step 3 of the SA [Roadmap for Easing COVID-19 Restrictions](http://www.covid-19.sa.gov.au/recovery). | SA Health, [COVID-19 update 29 June,](https://www.sahealth.sa.gov.au/wps/wcm/connect/public+content/sa+health+internet/about+us/news+and+media/all+media+releases/covid-19+update+29+june) media release, 29 June 2020. |
| 8 November, 2020 | SA government announced that in the state budget it would double its coronavirus economic stimulus package to AU$4 billion |  |
| 18 Nov, 2020 | A six-day lockdown from midnight that day was announced, ending 21 November due to misleading information provided to contract tracers. |  |
| 5 March 2021 | Oxford-AstraZeneca vaccine became available |  |
| 30 April 2021 | South Australia's first COVID-19 mass vaccination hub opened at Adelaide Showground |  |
