# Supplementary material for: Relationships between pre-pandemic mental health, sociodemographic factors and health behaviours in older adults during the acute onset of COVID-19 in Australia: A descriptive analysis
Source: PLoS One. 2026 Apr 23;21(4):e0346787. doi: 10.1371/journal.pone.0346787 (PMC13105359; doi:10.1371/journal.pone.0346787)
Supplement: S2 File — (DOCX) [file pone.0346787.s002.docx]

**S2. Complete questionnaire list**

**Change in work and financial position**

We determined change in work situation using the following items; “Work situation change due to COVID-19”, “How much effect has COVID-19 had on the following areas of your work life?”. Change in financial status due to the pandemic was determined with “As a result of COVID-19, has your financial position changed?”. Current income, including the Australian Federal Government COVID-19 financial response, was also asked. The response options for each question are provided in Supplement 2.

**Nutrition**

Questions regarding nutrition included “Since the coronavirus/COVID-19 pandemic began in March, is the overall amount of food that you eat …..”, *Much less than before COVID-19; Less than before COVID-19; Same as before COVID-19; Higher than before COVID-19; Much higher than before COVID-19; Don’t know*. Responses were recategorized to less than before, same as before, and higher than before.

**Neighbourhood safety and social support**

Neighbourhood safety was determined from the following statements “Do you feel that your neighbourhood is a safe place”, and “Do you feel people in your neighbourhood can be trusted” with response options including *all of the time; most of the time’ none/some of the time; Don’t know*.

As a measure of social support, we asked, “Can you get help from family, friends or neighbours when you need it?” and response options were combined as follows: *none of the time/some of time/don’t know; most of the time; all of the time*.

**Sleep quality**

Sleep quality specifically during the COVID-19 restrictions (i.e. during March and May) was assessed with the Pittsburgh Sleep Quality Index, with a score of ≥6 indicated poor sleep quality[16]. We also asked “How many nights a week did you have a problem with your sleep?” and “During restrictions, to what extent did poor sleep- “affect your mood, energy, or relationships?”; “affect your concentration, productivity, or ability to stay awake?”; “Troubled you in general?”. Responses included *not at all; a little; somewhat; much; very much.*

**Mastery**

Mastery was assessed using the following statement, “Since Covid-19, I have little control over things that happen to me”. Response items were summarised as *Strongly agree; agree; neutral; disagree; strongly disagree*.

**Behavioural risk factors for chronic disease**

Alcohol consumption was assessed using the National Drug Strategy Household Survey (NDSHS)[17] items. This specifies that for males and females, high risk alcohol consumption is defined as >10 drinks/week OR <10 drinks/week but ≥5 drinks on any day.

Physical activity was according to the Active Australia Survey[18] classifying as activity as sedentary, activity but less than 150 min/week and at least 150 min/week of walking, moderate activity or vigorous activity.

We also identified missed or delayed prescribed medication with “As a result of COVID-19, have you missed or delayed getting regular prescription medication (yes/no).

Finally, we asked “How confident are you with filling out medical forms by yourself? as an indicator of health literacy[19] . Final responses were categorised for analysis as *not at all; a little; moderately; Very; Extremely.*
