# Supplementary material for: Relationships between pre-pandemic mental health, sociodemographic factors and health behaviours in older adults during the acute onset of COVID-19 in Australia: A descriptive analysis
Source: PLoS One. 2026 Apr 23;21(4):e0346787. doi: 10.1371/journal.pone.0346787 (PMC13105359; doi:10.1371/journal.pone.0346787)
Supplement: S3 File — (DOCX) [file pone.0346787.s003.docx]

Supplementary Table 3. Sex-specific logistic regression analysis of factors associated with depression and anxiety symptoms at follow up.

|  | Males (n=598) | | | | | Females (n=375) | | | | | | | |  |  |  |  |  |  |  |  |  |
| --- | --- | --- | --- | --- | --- | --- | --- | --- | --- | --- | --- | --- | --- | --- | --- | --- | --- | --- | --- | --- | --- | --- |
|  | Model 1 |  | Model 2 | |  | Model 1 | | |  | Model 2 | | |  |  |  |  |  |  |  |  |  |  |
|  | OR (95% CI) | *p-value* | OR (95% CI) | | *p-value* | OR (95% CI) | | | *p-value* | OR (95% CI) | | | *p-value* |  |  |  |  |  |  |  |  |  |
| **Age, yr** |  |  |  | |  |  | | |  |  | | |  |  |  |  |  |  |  |  |  |  |
| 35-54y | 3.38 (1.46-7.83) | 0.004 | 3.45 (1.40-8.53) | | 0.007 | 1.65 (0.69-4.00) | | | 0.262 | 1.02 (0.38-2.74) | | | 0.975 |  |  |  |  |  |  |  |  |  |
| 55-64 | 1.02 (0.53-1.96) | 0.961 | 1.07 (0.53-2.20) | | 0.844 | 1.06 (0.51-2.20) | | | 0.876 | 0.75 (0.33-1.67) | | | 0.477 |  |  |  |  |  |  |  |  |  |
| ≥65 | 1.00 |  | 1.00 | |  | 1.00 | | |  | 1.00 | | |  |  |  |  |  |  |  |  |  |  |
| **Overall food intake/day since restrictions began** |  |  |  | |  |  | | |  |  | | |  |  |  |  |  |  |  |  |  |  |
| < before | 1.92 (0.77-4.79) | 0.164 | 2.46 (0.96-6.30) | | 0.060 | 2.84 (0.95-8.49) | | | 0.061 | 2.74 (0.85-8.81) | | | 0.091 |  |  |  |  |  |  |  |  |  |
| >before | 1.63 (0.74-3.59) | 0.228 | 1.25 (0.53-2.96 | | 0.606 | 2.28 (1.07-4.84) | | | 0.033 | 2.88 (1.28-6.49) | | | 0.011 |  |  |  |  |  |  |  |  |  |
| same | 1.00 |  | 1.00 | |  | 1.00 | | |  | 1.00 | | |  |  |  |  |  |  |  |  |  |  |
| **Smoking** |  |  |  | |  |  | | |  |  | | |  |  |  |  |  |  |  |  |  |  |
| yes/some days | 3.05 (1.10-8.45) | 0.031 | 2.51 (0.82-7.69) | | 0.108 | 2.12 (0.54-8.32) | | | 0.283 | 1.13 (0.24-5.27) | | | 0.880 |  |  |  |  |  |  |  |  |  |
| non-smoker | 1.00 |  | 1.00 | |  | 1.00 | | |  | 1.00 | | |  |  |  |  |  |  |  |  |  |  |
| **Missed/delayed getting regular prescription medication?** |  |  |  | |  |  | | |  |  | | |  |  |  |  |  |  |  |  |  |  |
| yes | 2.17 (0.93-5.06) | 0.074 | 1.34 (0.51-3.50) | | 0.557 | 2.62 (0.93-7.39) | | | 0.068 | 1.93 (0.61-6.11) | | | 0.263 |  |  |  |  |  |  |  |  |  |
| no | 1.00 |  | 1.00 | |  | 1.00 | | |  | 1.00 | | |  |  |  |  |  |  |  |  |  |  |
| **Financial stress** |  |  |  | |  |  | | |  |  | | |  |  |  |  |  |  |  |  |  |  |
| Spending equivalent to/exceeds earnings | 1.58 (0.76-3.27) | 0.217 | 2.41 (1.07-5.42) | | 0.034 | 1.69 (0.79-3.59) | | | 0.174 | 1.45 (0.63-3.35) | | | 0.384 |  |  |  |  |  |  |  |  |  |
| don’t know/refused | 0.51 (0.18-1.45) | 0.206 | 0.70 (0.24-2.00) | | 0.504 | 0.89 (0.34-2.36) | | | 0.819 | 1.08 (0.39-3.01) | | | 0.880 |  |  |  |  |  |  |  |  |  |
| saves a lot/ bit left over saved or spent | 1.00 |  | 1.00 | |  | 1.00 | | |  | 1.00 | | |  |  |  |  |  |  |  |  |  |  |
| **Confident filling medical forms alone** |  |  |  | |  |  | | |  |  | | |  |  |  |  |  |  |  |  |  |  |
| not at all/a little/moderate | 1.23 (0.63-2.41) | 0.537 | 1.24 (0.59-2.61) | | 0.569 | 1.15 (0.51-2.63) | | | 0.731 | 1.09 (0.44-2.71) | | | 0.860 |  |  |  |  |  |  |  |  |  |
| extremely/very | 1.00 |  | 1.00 | |  | 1.00 | | |  | 1.00 | | |  |  |  |  |  |  |  |  |  |  |
| **Social support (Can get help from family, friends or neighbours when needed)** |  |  |  | |  |  | | |  |  | | |  |  |  |  |  |  |  |  |  |  |
| none/some of time | 2.82 (1.37-5.83) | 0.005 | 3.00 (1.36-6.61) | | 0.007 | 3.83 (1.50-9.80) | | | 0.005 | 3.10 (1.12-8.62) | | | 0.030 |  |  |  |  |  |  |  |  |  |
| most of time | 1.47 (0.81-2.67) | 0.207 | 1.52 (0.79-2.89) | | 0.208 | 2.48 (1.28-4.84) | | | 0.007 | 2.28 (1.10-4.69) | | | 0.026 |  |  |  |  |  |  |  |  |  |
| all of the time | 1.00 |  | 1.00 | |  | 1.00 | | |  | 1.00 | | |  |  |  |  |  |  |  |  |  |  |
| **Mastery (since covid, have little control over things that happen to me)** |  |  |  | |  |  | | |  |  | | |  |  |  |  |  |  |  |  |  |  |
| neutral | 3.99 (2.16-7.37) | <0.001 | 4.06 (2.08-7.95) | | <0.001 | 3.60 (1.72-7.56) | | | 0.001 | 2.62 (1.16-5.92) | | | 0.021 |  |  |  |  |  |  |  |  |  |
| agree | 7.93 (3.82-16.4) | <0.001 | 6.34 (2.79-14.4) | | <0.001 | 8.30 (3.81-18.1) | | | <0.001 | 5.65 (2.40-13.3) | | | <0.001 |  |  |  |  |  |  |  |  |  |
| disagree | 1.00 |  | 1.00 | |  | 1.00 | | |  | 1.00 | | |  |  |  |  |  |  |  |  |  |  |
| **Sleep-During the restrictions, to what extent has poor sleep, troubled you in general?** |  |  |  | |  |  | | |  |  | | |  |  |  |  |  |  |  |  |  |  |
| somewhat/much/very much | 5.97 (3.27-10.9) | <0.001 | 3.84 (2.00-7.37) | | <0.001 | 3.46 (1.71-7.01) | | | 0.001 | 3.08 (1.44-6.62) | | | 0.004 |  |  |  |  |  |  |  |  |  |
| a little | 14.7 (6.63-32.5) | <0.001 | 8.38 (3.51-20.0) | | <0.001 | 12.3 (5.17-29.4) | | | <0.001 | 6.88 (2.66-17.8) | | | <0.001 |  |  |  |  |  |  |  |  |  |
| not at all | 1.00 |  | 1.00 | |  | 1.00 | | |  | 1.00 | | |  |  |  |  |  |  |  |  |  |  |
| **Previous depressive symptoms (2015-16)†** |  |  |  | |  |  | | |  |  | | |  |  |  |  |  |  |  |  |  |  |
| At least mild |  |  | 9.35 (4.95-17.7 | | <0.001 |  | | |  | 9.19 (4.24-19.9) | | | <0.001 |  |  |  |  |  |  |  |  |  |
| no symptoms |  |  | 1.00 | |  |  | | |  | 1.00 | | |  |  |  |  |  |  |  |  |  |  |
| **^†^** CES-D ≥16 for NWAHS or BDI-1A, ≥13 for FAMAS participants  Model 2: model 1 additionally adjusted for previous depressive symptoms. Of 711 male and 462 females available for analysis, 598 males and 375 females had data available  on previous depressive symptoms. | | | | | | | | | | | | | | | | | | | |  |  |  |
|  | | | |  | | |  |  | | |  |  | |  |  |  |  |  |  |  |  |  |
